# Plant characterization of genetically modified maize hybrids MON-89Ø34-3 × MON-88Ø17-3, MON-89Ø34-3 × MON-ØØ6Ø3-6, and MON-ØØ6Ø3-6: alternatives for maize production in Mexico

**DOI:** 10.1007/s11248-016-9991-z

**Published:** 2016-10-22

**Authors:** Oscar Heredia Díaz, José Luis Aldaba Meza, Baltazar M. Baltazar, Germán Bojórquez Bojórquez, Luciano Castro Espinoza, José Luis Corrales Madrid, Juan Manuel de la Fuente Martínez, Héctor Abel Durán Pompa, José Alonso Escobedo, Armando Espinoza Banda, José Antonio Garzón Tiznado, Juvencio González García, José Luis Guzmán Rodríguez, Jesús Ignacio Madueño Martínez, José Luis Martínez Carrillo, Chen Meng, Francisco Javier Quiñones Pando, Enrique Rosales Robles, Ignacio Ruiz Hernández, José Elías Treviño Ramírez, Hugo Raúl Uribe Montes, Francisco Zavala García

**Affiliations:** 1 Monsanto Company, 800 North Lindbergh Blvd, St. Louis, MO 63167 USA; 2 Facultad de Ciencias Agrícolas y Forestales, Universidad Autónoma de Chihuahua, Km. 2.5 Carr. Delicias-Rosales, Cd. Delicias, C.P. 33000 Chihuahua Mexico; 3 Facultad de Ciencias Químico Biológicas, Universidad Autónoma de Sinaloa, Ave. Las Américas y Josefa Ortiz, S/N Culiacán, C.P. 80000 Sinaloa Mexico; 4 Dirección de Recursos Naturales, Instituto Tecnológico de Sonora, 5 de Febrero 818 Sur, Colonia Centro Cd., Obregón, C.P. 85000 Sonora Mexico; 5 Facultad de Agronomía, Universidad Autónoma de Nuevo León, Av. Francisco Villa S/N, Col. Ex Hacienda “El Canadá”, Escobedo, C.P. 66050 Nuevo León Mexico; 6 Universidad Autónoma Agraria Antonio Narro, Unidad Laguna, Periférico Raúl López Sánchez y Carretera Santa Fe, Col. Valle Verde, Torreón, C.P. 27054 Coahuila Mexico

**Keywords:** Environmental risk assessment, Center of origin of maize, GM maize, Meta-analysis, Ecoregions, Data transportability

## Abstract

Environmental risk assessment (ERA) of genetically modified (GM) crops is a process to evaluate whether the biotechnology trait(s) in a GM crop may result in increased pest potential or harm to the environment. In this analysis, two GM insect-resistant (IR) herbicide-tolerant maize hybrids (MON-89Ø34-3 × MON-88Ø17-3 and MON-89Ø34-3 × MON-ØØ6Ø3-6) and one herbicide-tolerant GM hybrid (MON-ØØ6Ø3-6) were compared with conventional maize hybrids of similar genetic backgrounds. Two sets of studies, Experimental Phase and Pilot Phase, were conducted across five ecological regions (ecoregions) in Mexico during 2009–2013, and data were subject to meta-analysis. Results from the Experimental Phase studies, which were used for ERA, indicated that the three GM hybrids were not different from conventional maize for early stand count, days-to-silking, days-to-anthesis, root lodging, stalk lodging, or final stand count. Statistically significant differences were observed for seedling vigor, ear height, plant height, grain moisture, and grain yield, particularly in the IR hybrids; however, none of these phenotypic differences are expected to contribute to a biological or ecological change that would result in an increased pest potential or ecological risk when cultivating these GM hybrids. Overall, results from the Experimental Phase studies are consistent with those from other world regions, confirming that there are no additional risks compared to conventional maize. Results from Pilot Phase studies indicated that, compared to conventional maize hybrids, no differences were detected for the agronomic and phenotypic characteristics measured on the three GM maize hybrids, with the exception of grain moisture and grain yield in the IR hybrids. Since MON-89Ø34-3 × MON-88Ø17-3 and MON-89Ø34-3 × MON-ØØ6Ø3-6 confer resistance to target insect pests, they are an alternative for farmers in Mexico to protect the crop from insect damage. Additionally, the herbicide tolerance conferred by all three GM hybrids enables more cost-effective weed management.

**Electronic supplementary material:**

The online version of this article (doi:10.1007/s11248-016-9991-z) contains supplementary material, which is available to authorized users.

## Introduction

Mexico’s regulatory framework addresses the cultivation of genetically modified (GM) crops (Gutiérrez 2010). This framework consists of a Biosafety Law and an additional Bylaw published in 2005 and 2008, respectively (DOF 2005, 2008). The Biosafety Law requires stepwise field evaluations of GM crops, starting with small plots in an Experimental Phase followed by larger plots in a Pilot Phase prior to commercial plantings. Plant characterization data generated during the Experimental Phase allow Mexican regulators to assess for unintended effects and the absence of adverse impact of the GM crop on the receiving environment and plant health. Subsequently, these data facilitate the issuance of planting permits, thus advancing the regulatory process.

In-country data requirements by the Mexican regulatory system are of a confirmatory nature, given that the standard process to demonstrate crop safety for any commercial GM crop includes a stringent battery of rigorous scientific evaluations and independent regulatory reviews by most grain-importing and cultivating countries (Nakai et al. 2015). By the time a GM crop product is introduced into the Mexican regulatory system, extensive evaluation has already taken place in other world areas. These evaluations examine the potential for food, feed, and environmental risk. Phenotypic and agronomic characterization of GM crops relative to conventional crops provides a comparative context that is used within the natural variation of the crop to establish “familiarity” (Nickson and Horak 2006). Ultimately, this comparative assessment is used to assess potential risks that may be hypothesized regarding the cultivation of GM crops (Horak et al. 2007, 2015; Raybould et al. 2012; Sammons et al. 2014) and that are assessed on the basis of specific environmental protection goals (Nickson 2008). The concept of familiarity is useful to decision makers and regulators because it comes from preexisting general knowledge of the biology and agronomic characteristics of a crop, previous field cultivation results, expert opinions, historical agronomic experience, and the characteristics of the trait(s) introduced, the receiving environment, and their interactions (Nickson and Horak 2006). For example, Raybould et al. (2012) laid out a possible scenario by which an ecological harm may arise from cultivating GM crops when some of the seed produced is dispersed into a new environment, establishing new populations that may reduce the abundance of the original crop population or natural vegetation. In cases where maize is the receiving crop, familiarity would help dismiss this scenario because maize cannot survive outside of cultivation given that any weediness characteristics have been eliminated during the domestication and selection processes (Gould 1968; Keeler 1989; Martínez-Soriano et al. 2002).

Mexico is a “mega-diverse” country and is one of more than 17 nations that together contain nearly 70 % of the global diversity of plant and animal species (CONABIO 2009). Several ecoregions have been defined in Mexico based on biodiversity criteria (INEGI-CONABIO-INE 2008; Wiken et al. 2011). For conservation purposes, an ecoregion is defined as a large unit of land containing a geographically characteristic assemblage of species, natural communities, and environmental conditions. The boundaries of an ecoregion are not fixed and sharp, but rather encompass an area where important ecological and evolutionary processes generally interact. In contrast, field studies to characterize GM crops are typically implemented in areas devoted to agricultural production. These agricultural areas have relatively homogeneous characteristics (e.g., climate, soils, water availability, infrastructure) and are contained within the larger, usually more heterogenous, ecoregions. Field studies with GM plant materials are implemented under confinement conditions as a biosafety measure. There are no international standards for conducting confined field trials (CFTs), and national regulations and guidance vary by country with regard to trial design, number of sites, and duration (Garcia-Alonso et al. 2014). After almost two decades of cultivation of GM crops worldwide, a conceptual model and methodological scheme has been proposed in which it is possible to utilize data generated in one region to assess environmental risk for another region (Garcia-Alonso et al. 2014; Horak et al. 2015; Ahmad et al. 2016). Results from field studies obtained from multiple geographies for GM soybean (Horak et al. 2015) and GM maize (Nakai et al. 2015; Ahmad et al. 2016) demonstrate the utility of generating relevant data that are transportable across regions for the ERA of GM crops. This approach can be readily applied in practice when the assessment endpoints are demonstrably similar to those of other regions where new CFTs are being considered. Currently in Mexico, it is not possible to use data generated in one ecoregion for approvals in another. As described above, however, agricultural fields are typically located in disturbed environments that tend to be homogeneous even though they may be in different ecoregions.

GM crops have been grown commercially for more than 20 years. By 2014, 181.5 million hectares of GM crops were planted in 29 countries by more than 18 million growers (James 2014; Aldemita et al. 2015). In general, the rapid adoption of GM crops by farmers is due to their benefits such as higher yield potential, obtained by protecting against insects pests and weeds, and lower production costs (Areal et al. 2013; Solleiro Rebolledo and Castañón Ibarra 2013). In both developed and developing countries, economic profits associated with GM crops are usually higher than those achieved with conventional varieties due to the combination of yield increases and reduction of production costs (Finger et al. 2011; Areal et al. 2013; Klümper and Qaim 2014). Insect-resistant (IR) crops increase farmers’ profits as a result of higher yields and lower expenditure on insecticides (Qaim 2009). Herbicide-tolerant (HT) crops facilitate implementation of more cost-effective and efficacious weed control programs by enabling application of broad-spectrum herbicides (Klümper and Qaim 2014).

The use of improved varieties and hybrids combined with appropriate agronomic practices has helped to reduce crop losses due to pests and diseases (Oerke 2006; Blanco et al. 2014; Vargas-Parada 2014). However, in spite of these improvements, yield losses of up to 31 % of maize production have been reported due to pests (insects, weeds) and diseases (Oerke 2006). In Mexico, where approximately 6–8 million hectares are planted to corn annually, loss in maize production due to insects, diseases, and other pests is estimated as high as 30 % (Oerke 2006). A recent survey in Mexico documented that 3000 tons of insecticide active ingredients are required each year to reduce damage by fall armyworm (*Spodoptera frugiperda* Smith), corn earworm (*Helicoverpa zea* Boddie), and cutworms (*Agrotis ipsilon* Hufnagel) (Blanco et al. 2014). Mexico’s current use of pesticides is the highest per unit area in North America, at 4.5 kg ha^−1^ compared to 2.2 and 1 kg ha^−1^ for the USA and Canada, respectively (Stokstad 2013). The task to increase maize production in Mexico requires the use and adoption of technologies and best practices of modern agriculture (Vargas-Parada 2014). These include conventional improved seed, GM IR and HT varieties with higher yield potential, and best management practices for the control of insects, pathogens, and weeds and tolerance to abiotic stresses (Oerke 2006; Vargas-Parada 2014). Furthermore, greater adoption of integrated pest management (IPM) systems combined with the development and availability of pest-resistant maize varieties should reduce the use of conventional pesticides and help Mexico to reduce annual corn grain imports (Blanco et al. 2014).

The objectives of this analysis were, first, to characterize and assess three maize GM hybrids—MON-89Ø34-3 × MON-88Ø17-3 (three stacked IR traits plus a single HT trait), MON-89Ø34-3 × MON-ØØ6Ø3-6 (two stacked IR traits plus a single HT trait), and MON-ØØ6Ø3-6 (single HT trait)—grown at several sites in Mexico for evidence of biologically meaningful agronomic and phenotypic differences or adverse ecological effects due to the introgression of IR and/or HT biotech traits (ERA). The second objective was to confirm biological efficacy in terms of plant response of MON-89Ø34-3 × MON-88Ø17-3 and MON-89Ø34-3 × MON-ØØ6Ø3-6 maize hybrids against lepidopteran and coleopteran insect pests, and to assess tolerance to glyphosate-based herbicides and efficacy of weed management programs by all three maize hybrids.

## Materials and methods

### Sites

**Fig. 1:**
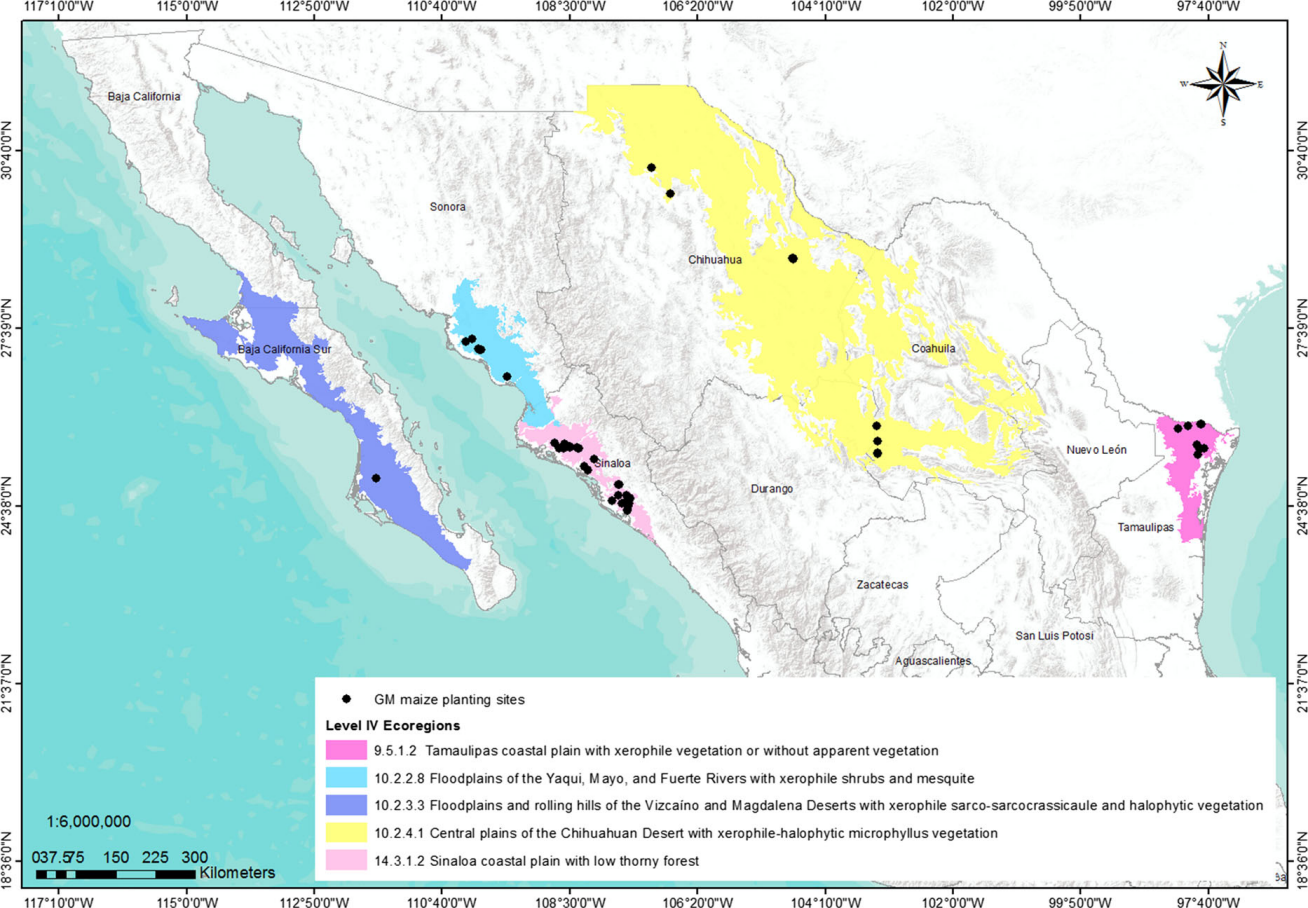
Map of level IV ecological regions (ecoregions) of Mexico where GM field studies were conducted during 2009–2013

Study sites in maize production areas were located within five ecoregions: (i) 9.5.1.2 = Tamaulipas coastal plain with xerophile vegetation or without apparent vegetation; (ii) 10.2.2.8 = floodplains of the Yaqui, Mayo, and Fuerte Rivers with xerophile shrubs and mesquite; (iii) 10.2.3.3 = floodplains and rolling hills of the Vizcaíno and Magdalena Deserts with xerophile sarco-sarcocrassicaule and halophytic vegetation; (iv) 10.2.4.1 = central plains of the Chihuahuan Desert with xerophile-halophytic microphyllus vegetation; and (v) 14.3.1.2 = Sinaloa coastal plain with low thorny forest (INEGI-CONABIO-INE 2008) (Supplementary Table 1, Fig. 1). Thirty-six studies, 19 Experimental Phase (smaller trials) and 17 Pilot Phase (larger trials), were conducted across maize production regions of the Mexican states of Sinaloa, Sonora, Chihuahua, Coahuila and Durango (Comarca Lagunera), Tamaulipas, and Baja California Sur during the 2009–2013 crop seasons (Supplementary Table 2).

### Test and control materials

The test materials were GM maize hybrids MON-89Ø34-3 × MON-88Ø17-3, MON-89Ø34-3 × MON-ØØ6Ø3-6, and MON-ØØ6Ø3-6, and the control materials were corresponding conventional (non-GM) isohybrids. Within each study, the three GM maize hybrids and the conventional maize control hybrid were all in the same hybrid (genetic background). At all but one site (Chihuahua), the hybrids were in a genetic background broadly adapted to the environmental conditions of northern Mexican states; at Chihuahua, an early-maturing hybrid background was used. GM hybrid MON-89Ø34-3 × MON-88Ø17-3 expresses three *Bt* proteins (Cry1A.105, Cry2Ab2, and Cry3Bb1) that confer resistance against aboveground lepidopteran insect pests and belowground local *Diabrotica* species. It also expresses the 5-enolpyruvylshikimate-3-phosphate synthase (EPSPS) protein, which confers tolerance to glyphosate herbicide. GM hybrid MON-89Ø34-3 × MON-ØØ6Ø3-6 expresses two *Bt* proteins (Cry1A.105 and Cry2Ab2) that confer resistance against aboveground lepidopteran insects pests, and also expresses the EPSPS protein. GM hybrid MON-ØØ6Ø3-6 expresses only the EPSPS protein.

### Trial implementation and crop management

Experimental Phase plot size ranged from 12 to 384 m^2^, and Pilot Phase plot size ranged from 398.7 to 4128 m^2^ (Supplementary Table 2). The main soil textures varied across locations and included clay, silty clay, clay loam, sandy loam, sandy clay loam, and sandy silt. Row spacing varied from 0.65 to 0.92 m, with a seeding rate of 5 to 10 seeds per meter and seed planting depth of 2 to 9 cm. Plot management was according to the recommendations by INIFAP-CIRNO for maize (Mendoza et al. 2003). Crop management practices included seedbed soil preparation, fertilization, irrigation, and insect and weed control according to regional best practices. Agronomic practices were conducted uniformly across all entries within a study in the Experimental Phase trials in order to eliminate an additional source of variation on the agronomic and phenotypic characteristics. However, in the Pilot Phase trials, insect and weed control practices were conducted according to each material’s phenotype, i.e., the IR/HT hybrids MON-89Ø34-3 × MON-88Ø17-3 and MON-89Ø34-3 × MON-ØØ6Ø3-6 GM did not require conventional insecticide applications for target lepidopteran insect pests, but MON-ØØ6Ø3-6 (HT only) and the conventional hybrid required two to four application of conventional insecticides to control lepidopteran pests across most sites. Weed control was also different between the GM hybrids (all HT) and the conventional control hybrid. Across all sites, one or two over-the-top applications of Faena Fuerte^®^ with Transorb^®^ 1 (540 g a.i. L^−1^), a glyphosate-containing herbicide, were made on the three GM hybrids at rates of 2 to 4 L ha^−1^. Weed control for the conventional control was mechanical (cultivator or manual) and/or by applications of selective herbicides. Subsequently, weed control was evaluated at approximately 11, 20, and 30 days after herbicide treatment in all of the studies.

### Experimental design, data collection, and analysis

GM maize hybrids MON-89Ø34-3 × MON-88Ø17-3, MON-89Ø34-3 × MON-ØØ6Ø3-6, and MON-ØØ6Ø3-6 and a corresponding conventional isohybrid control were planted in each of 36 studies (19 Experimental Phase, 17 Pilot Phase) in a randomized complete block design (RCBD) with three to four replications and up to four locations per ecoregion per year (Supplementary Tables 1 and 2). Twelve agronomic and phenotypic characteristics were evaluated throughout the season (Supplementary Table 3). In addition, plant response to target insect pests (i.e., stalk borer tunnel length and tunnel number, *Diabrotica* root damage, corn earworm damage, and *Spodoptera* leaf damage) were evaluated according to standard methods (Davis et al. 1992; Oleson et al. 2005). When present, cutworm (*Agrotis* and/or *Spodoptera* spp.) seedling damage was also documented (Supplementary Table 3). Weed control levels (% of total weed population eliminated) by total applications of glyphosate vs. mechanical weed control treatments were documented at multiple locations. Agronomic, phenotypic, and insect damage data collected from each individual study were subject to an analysis of variance (ANOVA). The means, standard errors, and sample sizes of the test and control hybrids for agronomic, phenotypic, and insect damage measurements from each study were generated from the statistical analyses of individual studies and included in a meta-analysis.

A meta-analysis uses standardized differences, i.e.,

1 $$d = (\bar{y}_{\text{test}} - \bar{y}_{\text{control}} )/s_{\text{p}}$$

where $\bar{y}_{\text{test}}$ and $\bar{y}_{\text{control}}$ are the sample mean of the test and control, respectively, and *s*_p_ is the pooled standard deviation, which is calculated from the individual test and control standard deviations. Thus, the standard errors were converted into standard deviations. The database spreadsheets were imported into the software Comprehensive Meta-Analysis™ (version 2, 2011; Biostat™, Englewood, NJ). Separate meta-analyses were conducted for each of the three test materials within each of the two regulatory phases (Experimental and Pilot) using random-effects models (Cochran and Cox 1957). In some cases, there were studies where the standard deviation for the test and/or the control was zero. These studies were excluded from the meta-analysis because *s*_p_ in Eq. (1) is assumed to be calculated from nonzero standard deviations. The meta-analysis used standardized differences from each individual study to compute a combined study effect. The combined study effect is a weighted standardized difference between test and control across studies, and the weights are functions of the sample sizes (Hedges and Olkin 1985). A random-effects model was used in each meta-analysis based on the assumption that the material effect interacts with the changing environments (sites), which is a common assumption in agronomic studies. The combined study effect obtained under the random-effects model is an estimate of the overall effect (standardized difference between test and control) across all potential environments. (The significance testing of this overall effect took the material-by-site interaction effect into account.) A 95 % confidence interval along with the *p* value was also obtained to describe the combined study effect. If the 95 % confidence interval contains zero, then the standardized difference favors neither the test nor the control. If the interval does not contain zero and is positive, then the standardized difference favors the test (test minus control is positive); if the interval does not contain zero and is negative, then the standardized difference favors the control (test minus control is negative). Statistical significance is defined by a *p* value <0.05. If the *p* value is less than 0.05, then the combined study effect is significantly different from zero, which means that the test and control are significantly different.

In addition, a regression analysis was implemented on yield data from IR hybrids MON-89Ø34-3 × MON-88Ø17-3 and MON-89Ø34-3 × MON-ØØ6Ø3-6 relative to the conventional control across all studies for which data were available. This analysis included sites that could not be included in the meta-analysis due to fewer than three replicates of data for traits analyzed. The numbers of studies included in the regression analysis were 32 Experimental Phase and 26 Pilot Phase studies.

## Results

Field evaluations of MON-89Ø34-3 × MON-88Ø17-3, MON-89Ø34-3 × MON-ØØ6Ø3-6, and MON-ØØ6Ø3-6 were well distributed and representative of each of the five ecoregions during 2009–2013 (Fig. 1). Together, the five ecoregions represent a wide range of conditions: an altitude range of 0–2400 meters above sea level; warm to semi-warm climate, with a mean annual temperature range of 17–26 °C; and subhumid to semiarid and very dry conditions with a mean annual rainfall range of 100–1069 mm (Supplementary Table 1). However, the lack of rainfall in semiarid and very dry environments had no impact on the crop growth because all trials were under irrigation and water was provided as needed (Mendoza et al. 2003), which is a typical practice during the autumn–winter season in these northern regions. Data from each of the 36 studies (Supplementary Table 2) were individually analyzed and a subsequent meta-analysis on the individual results of each trial was carried out. Meta-analysis grouped studies in comparative panels for the two regulatory phases (Experimental and Pilot) across the five ecoregions, multiple sites, and years (2009–2013).

### Agronomic and phenotypic characteristics

#### Experimental Phase studies

**Fig. 2:**
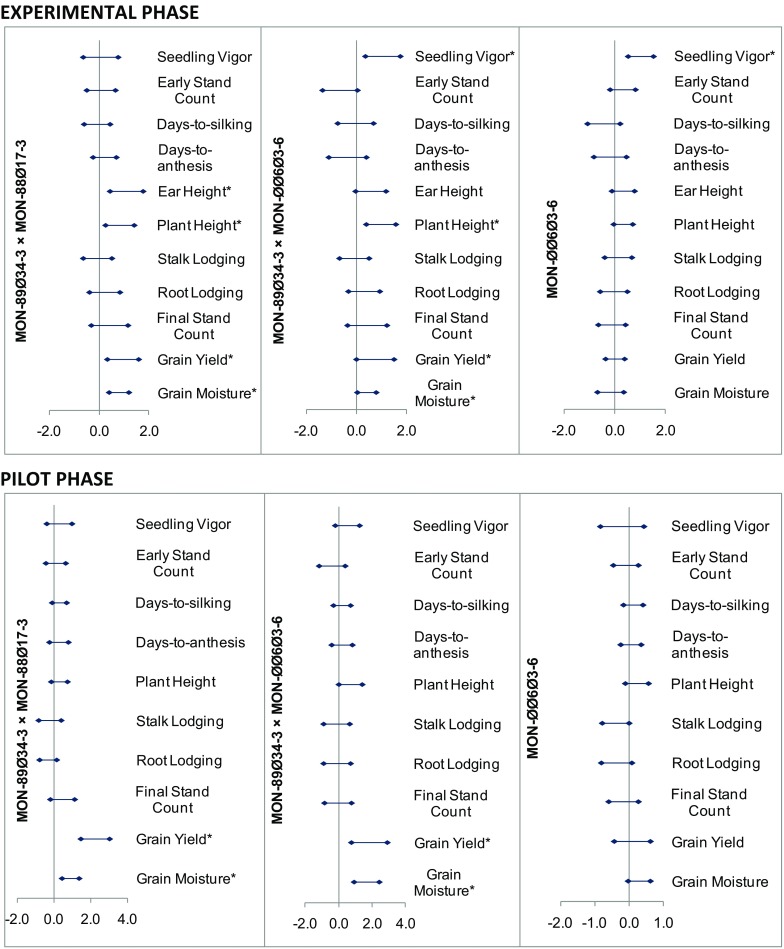
Agronomic and phenotypic combined study effect and confidence intervals of MON-89Ø34-3 × MON-88Ø17-3, MON-89Ø34-3 × MON-ØØ6Ø3-6, and MON-ØØ6Ø3-6 GM maize hybrids compared to the conventional control from Experimental Phase (*top*) and Pilot Phase (*bottom*) studies. Confidence intervals are shown as standardized differences (differences indicated in standard deviation units) derived from the meta-analysis. *Asterisks* indicate statistically significant differences between test and control at the 5 % level of significance

The results of the meta-analysis for the agronomic and phenotypic characteristics for Experimental Phase studies are presented in Fig. 2 (top), and the means and standard errors from individual analyses are presented in Table 1. Agronomic and phenotypic data were collected for 12 different variables across 15 Experimental Phase studies, except that ear height was analyzed in only 11 studies (Table 1). Data were analyzed individually and by meta-analysis to test for differences between the three GM maize hybrids and the conventional control for all variables except dropped ears, which had zero variability, preventing application of a statistical analysis. Thus, a total of 33 statistical tests were conducted between the three GM maize hybrids and the conventional control for 11 variables. No statistical differences were detected for early stand count, days-to-anthesis, days-to-silking, root lodging, stalk lodging, or final stand count for any of the GM hybrids compared to the conventional hybrid control (Table 1, Fig. 2). Furthermore, as noted above, no differences were observed for dropped ears between test and control entries as mean values were numerically low, with zero variability. Statistically significant differences (*p* ≤ 0.05) between MON-89Ø34-3 × MON-88Ø17-3 and the conventional hybrid control were found for ear height (108.2 vs. 103.3 cm), plant height (210.2 vs. 204.0 cm), grain moisture (18.8 % vs. 18.0 %), and grain yield (10.2 vs. 9.2 t ha^−1^). Statistically significant differences between MON-89Ø34-3 × MON-ØØ6Ø3-6 and the conventional hybrid control were detected for seedling vigor (7.6 vs. 6.6, on a scale of 1 = poor, 9 = best), plant height (208.2 vs. 201.7 cm), grain moisture (18.5 % vs. 18.1 %), and grain yield (10.0 vs. 9.1 t ha^−1^). The only statistically significant difference detected between MON-ØØ6Ø3-6 and the conventional control was seedling vigor (7.2 vs. 6.5). For MON-89Ø34-3 × MON-88Ø17-3 and MON-89Ø34-3 × MON-ØØ6Ø3-6 GM maize hybrids, which expressed both insect control (IR) and HT traits, plant height, grain moisture, and grain yield were the three characteristics in common that showed significantly higher values than the conventional hybrid control.

**Table 1:** Means and standard errors (SE) for phenotypic and agronomic characteristics of MON-89Ø34-3 × MON-88Ø17-3, MON-89Ø34-3 × MON-ØØ6Ø3-6, and MON-ØØ6Ø3-6 GM hybrids and the conventional control for Experimental Phase studies in Mexico during 2009–2013

Characteristic (unit)	Number of studies	Experimental Phase trials					
		MON-89Ø34-3 × MON-88Ø17-3		MON-89Ø34-3 × MON-ØØ6Ø3-6		MON-ØØ6Ø3-6	
		Mean (SE)		Mean (SE)		Mean (SE)	
		Test	Control	Test	Control	Test	Control
Seedling vigor (1 [poor] to 9 [best])	15	7.0 (0.3)	6.7 (0.2)	7.6 (0.3)*	6.6 (0.3)	7.2 (0.2)*	6.5 (0.2)
Early stand count	15	199.4 (35.0)	202.0 (36.0)	194.8 (35.5)	202.7 (36.0)	200.6 (32.5)	202.8 (36.0)
Days-to-silking	15	80.6 (5.4)	80.3 (5.3)	81.0 (5.4)	80.6 (5.3)	79.9 (5.2)	80.3 (5.3)
Days-to-anthesis	15	78.9 (5.3)	78.2 (5.1)	78.6 (5.2)	78.4 (5.1)	77.9 (5.0)	78.1 (5.0)
Ear height (cm)	11	108.2 (7.5)*	103.3 (7.4)	106.9 (8.0)	103.1 (7.4)	103.9 (7.8)	102.3 (7.6)
Plant height (cm)	15	210.2 (6.6)*	204.0 (6.5)	208.2 (7.5)*	201.7 (7.6)	204.8 (7.4)	202.7 (7.3)
Final stand count	15	109.6 (12.6)	103.3 (12.1)	108.6 (13.4)	102.8 (12.2)	103.0 (11.5)	103.0 (12.1)
Root lodging (%)	15	3.5 (1.7)	2.7 (1.1)	2.9 (1.1)	2.1 (0.8)	3.6 (2.0)	2.3 (0.8)
Stalk lodging (%)	15	2.7 (1.1)	4.1 (1.8)	3.7 (2.1)	4.0 (1.8)	4.0 (1.7)	4.0 (1.8)
Dropped ears^a^	15	0.04 (0.03)	0.08 (0.07)	0.02 (0.02)	0.12 (0.07)	0.07 (0.05)	0.08 (0.07)
Grain moisture (%)	15	18.8 (1.0)*	18.0 (0.9)	18.5 (1.0)*	18.1 (0.9)	18.0 (1.0)	18.1 (0.9)
Grain yield (t ha^−1^)	15	10.2 (0.6)*	9.2 (0.7)	10.0 (0.6)*	9.1 (0.8)	9.1 (0.8)	9.1 (0.8)

* Statistically significant at the 5 % level of significance

^a^Data on dropped ears were collected but not included in the meta-analysis due to lack of variability

#### Pilot Phase studies

The results of meta-analysis for agronomic and phenotypic characteristics for Pilot Phase trials are presented in Fig. 2 (bottom), and the means and standard errors from individual analyses are presented in Table 2. For comparisons between the three GM maize hybrids and the conventional control, mean values from up to 23 Pilot Phase studies were considered, depending on the evaluated GM hybrid and the observed agronomic/phenotypic characteristics (Table 2). The results of Pilot Phase studies indicated that the three GM maize hybrids were not statistically different from the control for the majority of characteristics evaluated (seedling vigor, early stand count, days-to-silking, days-to-anthesis, plant height, root lodging, stalk lodging, and final stand count; Fig. 2). Statistically significant differences between MON-89Ø34-3 × MON-88Ø17-3 and MON-89Ø34-3 × MON-ØØ6Ø3-6 GM maize hybrids and the conventional control (*p* ≤ 0.05) were detected for grain yield (8.0 vs. 6.6 t ha^−1^ and 7.9 vs. 6.5 t ha^−1^, respectively) and grain moisture (17.8 % vs. 16.8 % and 15.5 % vs. 13.7 %, respectively). The mean values for grain moisture and grain yield were higher for the GM maize hybrids in all comparisons (Table 2). In the Pilot Phase trials, no differences were detected between MON-ØØ6Ø3-6 and the conventional control for any of the characteristics measured.

**Table 2:** Means and standard errors (SE) for phenotypic and agronomic characteristics of MON-89Ø34-3 × MON-88Ø17-3, MON-89Ø34-3 × MON-ØØ6Ø3-6, and MON-ØØ6Ø3-6 GM hybrids and the conventional control for Pilot Phase studies in Mexico during 2009–2013

Characteristic (unit)	Pilot Phase trials								
	MON-89Ø34-3 × MON-88Ø17-3			MON-89Ø34-3 × MON-ØØ6Ø3-6			MON-ØØ6Ø3-6		
	Mean (SE)			Mean (SE)			Mean (SE)		
	Number of studies	Test	Control	Number of studies	Test	Control	Number of studies	Test	Control
Seedling vigor (1 [poor] to 9 [best])	10	4.7 (0.7)	4.5 (0.8)	6	6.5 (0.6)	5.8 (0.9)	10	4.4 (0.7)	4.5 (0.8)
Early stand count	21	474.2 (188.3)	474.1 (180.7)	14	490.5 (239.9)	535.2 (268.6)	20	501.2 (193.3)	494.4 (188.7)
Days-to-silking	23	81.0 (2.8)	80.4 (3.0)	16	79.0 (3.9)	78.1 (4.1)	22	80.6 (3.2)	80.9 (2.9)
Days-to-anthesis	23	79.3 (2.7)	78.7 (2.8)	16	77.5 (3.6)	76.6 (3.9)	22	78.6 (3.0)	79.2 (2.7)
Ear height^a^ (cm)	3	60.0 (9.7)	60.1 (9.2)	3	61.5 (9.2)	60.1 (9.2)	3	63.7 (14.8)	64.4 (13.4)
Plant height (cm)	21	191.8 (8.8)	189.6 (8.9)	14	177.1 (11.8)	173.3 (12.3)	20	190.5 (9.2)	189.0 (9.2)
Final stand count	20	472.4 (187.0)	461.9 (176.4)	13	451.7 (249.0)	487.0 (273.5)	19	484.3 (196.4)	473.2 (185.5)
Root lodging (%)	17	3.5 (1.5)	4.6 (2.0)	11	5.3 (3.3)	6.0 (3.4)	16	3.8 (1.9)	4.5 (2.1)
Dropped ears^a^	7	3.6 (3.2)	3.3 (2.7)	3	11.3 (10.0)	7.6 (6.1)	7	2.6 (2.3)	3.3 (2.7)
Stalk lodging (%)	19	1.8 (0.7)	2.2 (0.5)	13	1.3 (0.7)	1.5 (0.5)	18	1.5 (0.4)	2.1 (0.6)
Grain moisture (%)	17	17.8 (1.2)*	16.8 (1.1)	11	15.5 (1.0)*	13.7 (0.7)	16	17.0 (1.1)	16.6 (1.2)
Grain yield (t ha^−1^)	17	8.0 (0.7)*	6.6 (0.8)	11	7.9 (0.8)*	6.5 (1.1)	16	6.4 (0.7)	6.3 (0.8)

* Statistically significant at the 5 % level of significance

^a^Data on dropped ears and ear height were collected but not included in meta-analysis. Data on dropped ears were not included because of lack of variability; data on ear height were not included because of an insufficient number of studies for inclusion

As illustrated in Supplementary Fig. 1, there was a yield advantage in MON-89Ø34-3 × MON-88Ø17-3 and MON-89Ø34-3 × MON-ØØ6Ø3-6 GM maize hybrids relative to the conventional control when analyzed across all studies from both the Experimental and Pilot phases.

### Plant response to target insect pests—Experimental and Pilot Phase studies

The results of the combined study effects analysis for the insect damage measurements are presented in Supplementary Fig. 2, and the means and standard errors from individual analyses are presented in Table 3. Insect damage evaluations were documented in 5–19 Experimental Phase studies of MON-89Ø34-3 × MON-88Ø17-3 and MON-89Ø34-3 × MON-ØØ6Ø3-6 GM hybrids and the isohybrid conventional control (Table 3). The analysis across regions, sites, and years showed statistically significant differences (*p* ≤ 0.05) between MON-89Ø34-3 × MON-88Ø17-3 and the conventional control for *Diabrotica* root damage (0.05 vs. 0.11 rating), corn earworm damage (0.46 vs. 2.40 cm^2^), and *Spodoptera* leaf damage (0.20 vs. 1.94 rating) (Table 3 and Supplementary Fig. 2). MON-89Ø34-3 × MON-ØØ6Ø3-6 had statistically significant differences from the conventional control for corn earworm damage (0.31 vs. 1.87 cm^2^) and *Spodoptera* leaf damage (0.33 vs. 2.23 rating) (Table 3). As expected, in all cases of significant differences, the level of insect damage was greater for the conventional control using regional best practices for insect pest control than for the GM IR maize hybrids.

**Table 3:** Means and standard errors (SE) for insect damage characteristics of MON-89Ø34-3 × MON-88Ø17-3 and MON-89Ø34-3 × MON-ØØ6Ø3-6 GM hybrids and the conventional control in Experimental and Pilot Phase studies in Mexico during 2009–2013

Characteristic evaluated (unit)	Study type	MON-89Ø34-3 × MON-88Ø17-3			MON-89Ø34-3 × MON-ØØ6Ø3-6		
		No. of studies	Mean (SE)		No. of studies	Mean (SE)	
			Test	Control		Test	Control
Stalk borer (*Diatraea* spp.) tunnel length (cm)	Experimental	14	0.01 (0.01)	1.09 (0.57)	11	0.25 (0.24)	0.55 (0.31)
	Pilot	24	0.02 (0.01)	0.18 (0.07)*	11	0.00 (0.00)	0.17 (0.08)
Stalk borer (*Diatraea* spp.) tunnel number	Experimental	16	0.00 (0.00)	0.16 (0.06)	12	0.01 (0.01)	0.05 (0.01)
	Pilot	17	0.00 (0.00)	0.04 (0.01)*	4	0.01 (0.01)	0.10 (0.07)
*Diabrotica* root damage (0–3 scale)	Experimental	18	0.05 (0.01)	0.11 (0.02)*	5	0.13 (0.06)	0.12 (0.05)
	Pilot	26	0.01 (0.00)	0.02 (0.01)*	13	0.07 (0.04)	0.06 (0.04)
Corn earworm (*Helicoverpa zea* or *Spodoptera* spp.) damage (cm^2^)	Experimental	19	0.46 (0.18)	2.40 (0.51)*	15	0.31 (0.10)	1.87 (0.48)*
	Pilot	26	0.19 (0.10)	2.08 (0.31)*	13	0.11 (0.05)	1.91 (0.37)*
*Spodoptera* leaf damage (0–9 scale)	Experimental	19	0.20 (0.09)	1.94 (0.34)*	15	0.33 (0.14)	2.23 (0.41)*
	Pilot	16	0.06 (0.03)	1.49 (0.35)*	13	0.05 (0.03)	1.26 (0.41)
Cutworm^a^ (*Agrotis* or *Spodoptera* spp.) damage (number of seedlings)	Experimental	–	–	–	–	–	–
	Pilot	3	0 (0.00)	35.88 (25.45)*	3	0 (0.00)	37.11 (24.07)*

*Statistically significant at the 5 % level of significance

^a^Cutworm (*Agrotis* spp.) was not present in Experimental Phase trials, and was not included in meta-analysis for Pilot Phase trials because of the small number of studies

Insect damage evaluations were performed in 3–26 Pilot Phase studies on the two GM IR hybrids and the conventional control (Table 3, Supplementary Fig. 2). Results from the comparative analysis between the GM hybrid MON-89Ø34-3 × MON-88Ø17-3 and the conventional control showed statistically significant differences (*p* ≤ 0.05) for stalk borer tunnel length (0.02 vs. 0.18 cm), stalk borer tunnel number (0.00 vs. 0.04), *Diabrotica* root damage (0.01 vs. 0.02 rating), corn earworm damage (0.19 vs. 2.08 cm^2^), *Spodoptera* leaf damage (0.06 vs. 1.49 rating) and cutworm (*Agrotis* and/or *Spodoptera* spp.) damage (0.00 vs. 35.88 seedlings) (Table 3, Supplementary Fig. 2). MON-89Ø34-3 × MON-ØØ6Ø3-6 showed statistically significant differences (*p* ≤ 0.05) for corn earworm damage (0.11 vs. 1.91 cm^2^) and cutworm (*Agrotis* and/or *Spodoptera* spp.) damage (0.00 vs. 37.11 seedlings) (Table 3).

### Herbicide tolerance and weed control

Weed populations present in the three GM maize hybrids and the conventional isohybrid control were documented in Experimental Phase (2010–2012) and Pilot Phase (2012–2013) studies. The weed inventories included species adapted to the different regions and are typical of agricultural fields (Agundis Mata and Concepción Rodríguez 1978; Rosales Robles and Sánchez de la Cruz 2010). Only those weeds of agronomic importance are reported here. Twenty-five different weed species were documented in the Experimental Phase studies; the five most common species of agronomic interest were *Amaranthus palmeri*, *Chenopodium album*, *Convolvulus arvensis*, *Echinochloa colona*, and *Portulaca oleracea*. In the Pilot Phase studies, 48 different weed species were documented; the 13 species of most agronomic importance were *Amaranthus palmeri*, *Chamaesyce maculate*, *Chenopodium* spp., *Convolvulus arvensis*, *Cyperus esculentus*, *Echinochloa colona*, *Helianthus annuus*, *Leptochloa filiformis*, *Malva parviflora*, *Physalis* spp., *Portulaca oleracea*, *Solanum elaeagnifolium*, and *Sorghum halepense.* Overall, Sinaloa, Tamaulipas, and Comarca Lagunera showed the highest numbers of species, with 30, 20, and 13 different weed species, respectively.

Weed control evaluations were available from 4 to 8 Experimental Phase studies and from 9 to 16 Pilot Phase studies (Table 4) but were not subject to meta-analysis. Evaluations of weed control efficacy by glyphosate applications in the three GM hybrids and by mechanical treatment in the conventional isohybrid were conducted at 11, 20, and 30 days after treatment. Weed control in the GM hybrids with up to two over-the-top complete applications of Faena Fuerte^®^ with Transorb^®^ was consistently higher than for the alternative weed control treatment in the isohybrid control. On average, weed control in the GM hybrids was 3.1 % higher than in the isohybrid conventional control in the Experimental Phase studies, and 13.1 % higher than the control in the Pilot Phase studies (Table 4).

**Table 4:** Weed control means in Experimental and Pilot Phase studies of MON-89Ø34-3 × MON-88Ø17-3, MON-89Ø34-3 × MON-ØØ6Ø3-6, and MON-ØØ6Ø3-6 GM hybrids and the conventional isohybrid control in Mexico 2010–2013^a^

Hybrid	Average no. of days after treatment	Experimental			Pilot		
		No. of studies	% weed control		No. of studies	% weed control	
			Test	Control		Test	Control
MON-89Ø34-3 × MON-88Ø17-3	11		90.9	83.3		89.8	65.4
	20	8	95.9	95.2	16	92.8	77.9
	30		98.6	91.1		95.1	85.5
Average			95.1	89.9		92.5	76.3
MON-89Ø34-3 × MON-ØØ6Ø3-6	11		88.4	84.8		89.8	82.7
	20	8	97.5	99.7	9	95.6	86.4
	30		99.3	100.0		96.7	89.7
Average			95.0	94.9		94.0	86.3
MON-ØØ6Ø3-6	11		88.2	83.3		91.2	72.8
	20	4	96.8	95.2	15	94.9	77.9
	30		95.6	91.1		95.5	85.4
Average			93.5	89.9		93.9	78.7

^a^Weed control data were not subject to meta-analysis

## Discussion

Maize is the most important staple crop in Mexico, where a variety of maize types and production systems are present. Currently, in-country maize production is not sufficient to meet internal demand (Turrent Fernández et al. 2012; Blanco et al. 2014). Maize production is highly technified in Northern Mexico, and yield potentials are similar to those observed in the US Corn Belt (Turrent Fernández et al. 2012). Higher yields in Northern Mexico are associated with the use of improved conventional maize hybrids, irrigation, fertilizers, and appropriate crop management (e.g., weed and insect control). The use of pesticides in Mexico is the highest (4.5 kg ha^−1^) in North America (Stokstad 2013). Recent reports have documented the need and potential benefits of adopting IPM programs and newer, more sustainable technologies in corn production in Mexico (Bell et al. 2012; Blanco et al. 2014). IPM programs would minimize economic losses and would lower environmental and health risks. Once approved, GM maize potentially offers an additional tool for Mexican farmers for insect and/or weed control, increasing yields while reducing the number of insecticide applications.

### Environmental safety of GM maize hybrids in Mexico

The Mexican Biosafety Bylaw provides an operational guide for the preparation, review, and approval of GM crops in Mexico (DOF 2008). Current regulations require developers to present an ERA along with submission of experimental field trial applications. Apart from biosafety measures and administrative requirements, planting permits impose mandatory field protocols to generate in-country data to test for potential changes in GM crops that may be harmful to the environment, the plant, or animal health. These protocols have been conducted to advance the introduction of GM traits in maize cultivation systems in Northern Mexico. An ERA and the information generated from field studies of GM maize hybrids together enable regulators and agricultural policy makers to make informed decisions on the legal use and responsible adoption of GM crops in Mexico. Field studies with GM plant materials are conducted under robust and extensive biosafety protocols adopted by technology developers (industry and academic scientists) to ensure best management practices and extended life for GM crops (ETS 2015). In addition, studies comparing GM hybrids and controls in the same hybrid background provide a very powerful tool to minimize sources of variability and allow appropriate comparisons in order to best assess the potential environmental risks of introgressed GM traits.

Meta-analysis of agronomic and phenotypic characteristics from up to 15 Experimental Phase studies (33 statistical tests; Table 1) confirmed that MON-89Ø34-3 × MON-88Ø17-3, MON-89Ø34-3 × MON-ØØ6Ø3-6, and MON-ØØ6Ø3-6 GM maize hybrids were no different from the conventional hybrid control for early stand count, days-to-anthesis, days-to-silking, root lodging, stalk lodging, and final stand count, so phenotypic characteristics that define crop establishment (e.g., early stand count) were similar in both test and control materials (Fig. 2, top). Also, the absence of differences in the time to reach flowering (anthesis and silking) indicates that plant growth and development were similar between test and control materials and responded in a similar manner to growing conditions (temperature, soil moisture, nutrients, etc.) and crop management. In contrast, differences in grain yield between MON-89Ø34-3 × MON-88Ø17-3 and MON-89Ø34-3 × MON-ØØ6Ø3-6 GM maize hybrids and the conventional hybrid control (Fig. 2) could be a result of less damage induced by target insect lepidopteran pests (*Spodoptera* spp.), better plant health, and overall less stressful conditions for the GM hybrids, enabling them to reach more of their full yield potential.

Similarly, analysis from up to 23 Pilot Phase studies (Table 2) indicated that the three GM maize hybrids were not statistically different from the control for seedling vigor, early stand count, days-to-silking, days-to-anthesis, plant height, root lodging, stalk lodging, or final stand count (Fig. 2, bottom), confirming agronomic equivalence in key phenotypic and agronomic characteristics. Statistically significant differences between MON-89Ø34-3 × MON-88Ø17-3 and MON-89Ø34-3 × MON-ØØ6Ø3-6 GM maize hybrids and the conventional control (*p* ≤ 0.05) were detected for grain yield and grain moisture (Table 2). Higher grain moisture at harvest and higher grain yield were likely due to overall better plant health throughout the crop season, as a result of protection from target insect pests and better weed control through use of broad-spectrum herbicide applications. Regression analyses of these two hybrids indicated that in most cases they had greater yield than the conventional control hybrids across the Experimental and Pilot Phase studies (Supplementary Fig. 1). Furthermore, according to this analysis, growers would benefit from the use of IR/HT GM hybrids under conditions with relatively low yield potential. The average difference of 1.18 t ha^−1^ between GM IR maize hybrids and the conventional (non-GM) control, which was obtained using best regional management practices across the Experimental and Pilot studies, could represent a substantial increase in maize production in the northern states of Mexico.

Increased pest potential for a GM crop plant could include increased weediness in a cultivated field or increased invasiveness in natural vegetation. For a corn plant to become more weedy or invasive it would likely need seed dormancy and seed dispersal mechanisms to secure survival in new areas. No differences were detected in early stand count (Tables 1 and 2) or in laboratory seed germination tests, which showed >94 % germination in each of the hybrids (data not shown), thus indicating no changes in seed dormancy. Furthermore, no differences were observed for dropped ears (which would facilitate seed dispersal) between test and control entries, providing additional evidence that the GM traits have not increased weediness (Tables 1 and 2).

When the above results are considered in the context of an ERA and familiarity with the maize crop, none of the characteristics where statistically significant differences were found are considered to increase pest potential or any other potential risk to the receiving environment, plant health, or animal health.

### Economic and IPM benefits of GM maize hybrids

Many studies have documented the economic benefits to growers from the adoption of GM crops (Finger et al. 2011; Brookes and Barfoot 2015). Specifically, benefits included production cost savings (fewer pesticide applications), higher yields due to crop protection against targeted lepidopteran and coleopteran insect pests, and an overall improvement in the economics of farming households that have adopted GM crops (Finger et al. 2011; Lee et al. 2012; Areal et al. 2013; James 2014; Klümper and Qaim 2014). Relative increases in yield and profits from cultivating GM crops have been shown to be higher in developing countries than in developed countries (Finger et al. 2011; James 2014; Klümper and Qaim 2014). In Argentina, the benefit to farmers derived from adoption of GM IR maize hybrids was a net increase in yields of about 5 %, achieved by preventing losses caused by *Diatraea saccharalis* (stalk borer) and *Spodoptera frugiperda* (fall armyworm), and was estimated at US$170 M for the period 1998–2003 (Trigo 2011). The economic benefit in terms of cost reduction was US$20 ha^−1^ when using maize hybrids with stacked traits for IR and HT (Trigo 2011). Most of the cost reduction was likely due to decreases in insect control costs, with some additional savings due to reduced weed control costs. Other studies have confirmed such benefits (Qaim 2009; Solleiro Rebolledo and Castañón Ibarra 2013).

The threshold level (5 % plants damaged) to trigger lepidopteran pest control applications was never reached for the MON-89Ø34-3 × MON-88Ø17-3 and MON-89Ø34-3 × MON-ØØ6Ø3-6 GM maize hybrids in any study. This was expected given the intrinsic lepidopteran and coleopteran IR traits in these two hybrids conferred by the expression of *Bt*-derived proteins in the plant. As expected, no differences were detected between MON-ØØ6Ø3-6 and the conventional isohybrid maize for insect damage since neither plant material contains IR traits; both were affected equally by lepidopteran or coleopteran insect pests and both received chemical insect-control applications in these trials. Consequently, the threshold level (5 % plants damaged) to apply an insect-control treatment was reached once or twice during the growing season for MON-ØØ6Ø3-6 and the conventional isohybrid controls. These observations are consistent with those by Finger et al. (2011), Areal et al. (2013), and Blanco et al. (2014), thus confirming that GM IR and HT maize hybrids could be utilized as a beneficial alternative tool in IPM programs in Mexico. The reduction in use of additional insecticide applications to control lepidopteran pests such as fall armyworm (*Spodoptera* spp.) would help reduce the amount of insecticide active ingredients used per year in Mexico and would result in production cost savings for growers. In addition, use of GM IR hybrids may provide benefits associated with the reduction of pesticide loads in the environment.

These desirable outcomes from adopting GM IR crops have already been achieved by GM cotton growers in Mexico (Traxler and Godoy-Avila 2004) and maintained over the last 18 years (Brookes and Barfoot 2015). Genetically modified IR cotton varieties have resulted in more than 50 % reduction in pesticide use and have doubled the annual net revenue per hectare compared to that of growers cultivating conventional varieties in the Comarca Lagunera region (Traxler and Godoy-Avila 2004).

A popular agronomic practice in GM crop production systems in Argentina and the USA is reduced tillage (Trigo 2011; Lee et al. 2012). In Argentina, reduced tillage has made it possible to reverse the negative consequences related to extensive use of arable land and has allowed a more efficient energy use balance for crop production (Trigo 2011). In this context, the use of GM maize hybrids tolerant to the herbicide glyphosate has simplified weed control and/or reduced the costs involved (Norsworthy and Frederick 2005). Furthermore, implementation of reduced tillage practices may provide additional benefits including improved soil fertility, reduced erosion, increased carbon sequestration, and the use of herbicides with better environmental profiles (Trigo 2011). Similar results were observed in the present studies, where the three GM maize hybrids enabled over-the-top applications of Faena Fuerte^®^ with Transorb^®^ and provided better and more cost-effective weed control than the conventional control hybrids.

### Transportability of environmental risk assessment data

The agroecological characteristics of the level IV ecoregions where the studies were conducted represented a diverse range of environments suitable for agricultural utilization (Supplementary Tables 1 and 2). The main soil textures found across locations were clay, silty clay, clay loam, sandy loam, sandy clay loam, and sandy silt, with climates ranging from semi-warm (e.g., ecoregion 10.2.4.1, Chihuahua and Comarca Lagunera, with annual temperatures of 17–20 °C), to warm (e.g., ecoregion 14.3.1.2, Sinaloa, with annual temperatures of 22–26 °C). Rainfall was more variable, but most ecoregions received less than 200 mm with the exception of ecoregion 9.5.1.2 (Tamaulipas), with annual rainfall of 1069 mm. Likewise, the altitude was less than 400 m above sea level for most of the regions. However, ecoregion 10.2.4.1 (Chihuahua) had an altitude that ranged from 1000 to 2400 m above sea level. As previously mentioned, however, even geographically distinct ecoregions show homogeneity in factors such climate, soils, and water availability that define geographic zones within these ecoregions that are well suited for agricultural production and are used for this purpose. The similarity in agroclimatic characteristics of the agricultural sites where CFTs were implemented (Supplementary Table 1), in addition to the consistent results observed across all 36 studies through the use of standardized protocols, measurement endpoints, and data recording methods (Tables 1, 2, 3, 4), supports transportability of characterization data and assessments among ecoregions for ERA purposes. Overall, results from these ERA studies are consistent with other studies previously done in other world regions (US EPA 2008; James 2014). Furthermore, additional results of field studies obtained from multiple geographies for GM soybean (Horak et al. 2015) and GM maize (Nakai et al. 2015; Ahmad et al. 2016) demonstrate the utility of relevant data transportable across regions for the ERA of GM crops.

The Mexican regulatory framework requires an ERA to be submitted with each application for a CFT of a GM crop product and requires the implementation of plant characterization studies to test risk hypotheses on a case-by-case approach. To improve efficiency in the field testing efforts, representative and strategic field locations with similar agroecological characteristics across ecoregions are recommended to obtain results that would be transportable from one ecoregion to another (Garcia-Alonso et al. 2014). In general, application of data transportability conceptual models and procedures could increase the efficiency and power of field testing programs while reducing regulatory workload and costs. In the case of GM maize, ERAs done in Uruguay (CAI 2011, 2012) and Argentina (CONABIA, unpublished data) concluded that the risk of production and use of IR/HT GM maize for food and feed was not different from that of the conventional counterpart. In Argentina, this conclusion was based on data from plant characterization studies executed in US locations with very similar agroecological conditions to maize production regions in Argentina; therefore, the conclusions obtained from the US studies were transportable to the local agroecosystems (Monsanto Company internal communication). These approaches should be extended in the future.

## Conclusions

The regulatory framework in Mexico enabled field characterization of GM maize hybrids resistant to lepidopteran and/or coleopteran insects and tolerant to glyphosate herbicide in multiple locations of Northern Mexico in order to inform the ERA. The agronomic and phenotypic characteristics measured in 36 studies conducted across agricultural regions during 2009–2013 demonstrated that MON-89Ø34-3 × MON-88Ø17-3, MON-89Ø34-3 × MON-ØØ6Ø3-6, and MON-ØØ6Ø3-6 GM maize hybrids were not different from conventional maize hybrids in potential risks of weediness, pest potential, competition, or displacement. Thus, it was demonstrated that introgression of GM traits into the maize crop did not cause unexpected modifications to the plant or changes in the crop that would suggest changes in pest potential. Together, these results and crop management considerations to reduce gene flow (Baltazar et al. 2015) strongly support the conclusion that commercial plantings of GM maize hybrids would not increase potential environmental risks for cultivation and conservation of maize in Mexico. Furthermore, the results from these studies indicate that MON-89Ø34-3 × MON-88Ø17-3, MON-89Ø34-3 × MON-ØØ6Ø3-6, and MON-ØØ6Ø3-6 GM hybrids are valuable options for integrated crop production systems that can increase productivity per unit area, provide economic gains to Mexican farmers through reduction of production costs for pest control (insects and/or weeds), and benefit the environment.

## Electronic supplementary material

Below is the link to the electronic supplementary material.

Supplementary material 1 (PDF 177 kb)

## Footnotes

1 Registered trademark of Monsanto Technology LLC. Equivalent to Roundup Ultra^®.^
