# Supplementary material for: Plant characterization of genetically modified maize hybrids MON-89Ø34-3 × MON-88Ø17-3, MON-89Ø34-3 × MON-ØØ6Ø3-6, and MON-ØØ6Ø3-6: alternatives for maize production in Mexico
Source: Transgenic Res. 2016 Oct 22;26(1):135–51. doi: 10.1007/s11248-016-9991-z (PMC5243880; doi:10.1007/s11248-016-9991-z)
Supplement: Supplementary file 1 — Supplementary material 1 (PDF 177 kb) [file 11248_2016_9991_MOESM1_ESM.pdf]

Electronic Supplementary Material

Plant characterization of genetically modified maize hybrids MON-89Ø34-3 × MON-88Ø17-3, MON-89Ø34-3 × MON-ØØ6Ø3-6, and MON-ØØ6Ø3-6: alternatives for maize production in Mexico

Oscar Heredia Díaz[1]*, José Luis Aldaba Meza[2], Baltazar M. Baltazar[1], Germán Bojórquez Bojórquez[3], Luciano Castro Espinoza[4], José Luis Corrales Madrid[3], Juan Manuel de la Fuente Martínez[1], Héctor Durán Pompa[5], José Alonso Escobedo[6], Armando Espinoza Banda[6], José Antonio Garzón Tiznado[3], Juvencio González García[2], José Luis Guzmán Rodríguez[5], Jesús Ignacio Madueño Martínez[3], José Luis Martínez Carrillo[4], Chen Meng[1], Francisco Javier Quiñones Pando[2], Enrique Rosales Robles[6], Ignacio Ruiz Hernández[4], José Elías Treviño[5], Hugo Raúl Uribe Montes[2] and Francisco Zavala García[5]

[1]Monsanto Company, 800 North Lindbergh Blvd, St. Louis, MO 63167, USA
[2]Universidad Autónoma de Chihuahua, Facultad de Ciencias Agrícolas y Forestales, Km. 2.5 Carr. Delicias-Rosales, Cd. Delicias, Chihuahua C.P. 33000, México
[3]Universidad Autónoma de Sinaloa, Facultad de Ciencias Químico Biológicas, Ave. Las Américas y Josefa Ortiz, S/N Culiacán, Sinaloa, C.P. 80000, México
[4]Instituto Tecnológico de Sonora, Dirección de Recursos Naturales, 5 de Febrero 818 Sur, Colonia Centro Cd. Obregón, Sonora, C.P. 85000, México
[5]Universidad Autónoma de Nuevo León, Facultad de Agronomía, Av. Francisco Villa S/N, Col. Ex Hacienda "El Canadá", Escobedo, Nuevo León, C.P. 66050, México
[6]Universidad Autónoma Agraria Antonio Narro, Unidad Laguna, Periférico Raúl López Sánchez y Carretera Santa Fe, Col. Valle Verde, Torreón, Coahuila, C.P. 27059, México

*Corresponding author (oscar.heredia@monsanto.com)

Supplementary Table 1 Agroecological characteristics of each ecoregion where Experimental and/or Pilot Phase regulatory studies were conducted in Mexico during 2009–2013

Ecoregion[a]	Site ID	Climate description	Mean temperatures (°C)			Mean annual rainfall (mm)	Altitude (masl)	Wind speed (km/h)
			Annual	Min	Max			
9.5.1.2 Tamaulipas coastal plain with xerophile vegetation or without apparent vegetation	TAHU, TAVA, TAVH, TAVH2, TAMPS_21, TAMPS_28, TAMPS_15, TAMPS_21	Warm, semiarid continental portion; subhumid, semi-warm coastal portion	20 to 25	−2 to 0	36 to 38	1,069	0 to 200	130 to 160
10.2.2.8 Floodplains of the Yaqui, Mayo, and Fuerte Rivers, with xerophile shrubs and mesquite	LO, MF, MG, BASO, SOCO, SON_12, SON_02	Warm, very dry coast	19 to 25	0 to 2	38 to 42	206	0 to 200	160 to 190
10.2.3.3 Floodplains and rolling hills of the Vizcaíno and Magdalena Deserts with xerophile sarco-sarcocrassicaule and halophytic vegetation	BCSC	Semi-warm, very dry	20 to 22	2 to 4	36 to 44	100 to 200	0 to 600	130 to 190
10.2.4.1 Central plains of the Chihuahuan Desert with xerophile-halophytic microphyllus vegetation	CHIH1, CHIH2, LALA1, LALA2, CHIH_03, CHIH_18, LAG_12, LAG_09	Semi-warm, very dry	17 to 20	−10 to −2	38 to 42	340	1000 to 2400	130 to 190
14.3.1.2 Sinaloa coastal plain with low thorny forest	LF, SM, SILM, SIPE, SIVJ, SIAG, SICL, SIGU, SIN_72, SIN_77, SIN_81, SIN_82	Warm, very dry in Mochis region; warm and arid in other areas	22 to 26	5 to 8	36 to 42	125 to 600	200 to 400	130 to 220

[a] Level IV ecoregions as defined by INEGI-CONABIO-INE (2008)

Supplementary Table 2 Sites where MON-89Ø34-3 × MON-88Ø17-3, MON-89Ø34-3 × MON-ØØ6Ø3-6, and MON-ØØ6Ø3-6 GM maize hybrids and a conventional hybrid control were evaluated in Mexico during 2009–2013

Ecoregion[a]	Field/state	Site ID[b]	Planting date	Soil texture	Row spacing (m)	Seed depth (cm)	Seeding rate (seed/m)	Plot size (m^2)
Experimental Phase studies[c]								
14.3.1.2	Lote Fontes, Sin.	LF	8- to 9-Nov-09	Clay	0.75	5	9	105.0
14.3.1.2	Santa Marta, Sin.	SM	9- to 10-Nov-09	Clay	0.75	5	9	105.0
10.2.2.8	Block 1411, Son.	LO	30-Oct-09	Clay	0.80	5	5	100.8
10.2.2.8	Block 625, Son.	MF	2-Nov-09	Silty clay	0.80	5	5	100.8
10.2.2.8	Block 313, Son.	MG	31-Oct-09	Clay	0.80	5	5	100.8
9.5.1.2	Predio 4, Tam.	TAHU	2-Feb-10	Clay	0.82	5–6	10	114.8
9.5.1.2	Predio 7, Tam.	TAVA	14-Feb-10	Clay	0.82	5–6	10	114.8
14.3.1.2	Los Mochis, Sin.	SILM	16-Feb-11	Clay	0.80	2–3	7	128.0
14.3.1.2	Pericos, Sin.	SIPE	1-Mar-11	Clay	0.80	2–3	7	128.0
10.2.2.8	Bacum, Son.	BASO	19-Mar-11	Clay loam	0.80	2–3	7	64.0
10.2.2.8	Cd. Obregon, Son.	SOCO	5-Mar-11	Clay	0.80	2–3	7	96.0
10.2.4.1	Ahumada, Chih.	CHIH1	7-Jul-11	Sandy clay loam	0.92	5	8	14.4
10.2.4.1	Julimes, Chih.	CHIH2	9-Jul-11	Sandy loam	0.81	5	6	12.8
10.2.4.1	Fco. I Madero, Coah.	LALA1	21-Jul-11	Sandy clay loam	0.75	6.5	7	12.0
10.2.4.1	Matamoros, Coah.	LALA2	23-Jul-11	Sandy loam	0.75	6.5	7	12.0
9.5.1.2	Valle Hermoso1, Tam.	TAVH	18-Mar-12	Silty clay	0.80	6–8	8	384.0
9.5.1.2	Valle Hermoso2, Tam.	TAVH2	19-Mar-12	Sandy silt	0.80	6–8	8	384.0
14.3.1.2	Navolato, Sin.	SIVJ	25-Mar-12	Clay	0.80	6–8	8	24.0
10.2.3.3	Cd. Constitution, BCS	BCSC	28-Mar-12	Clay loam	0.75	6–8	8	84.0

Ecoregion[a]	Field/state	Site ID[b]	Planting date	Soil texture	Row spacing (m)	Seed depth (cm)	Seeding rate (seed/m)	Plot size (m^2)
Pilot Phase studies[c]								
14.3.1.2	Aguaruto, Sin.	SIAG	11-Feb-12	Clay	0.75	6–8	6	1,020.0
14.3.1.2	Culiacancito, Sin.	SICL	27-Jan-12	Clay loam	0.75	6–8	6	990.0
14.3.1.2	Guamuchil, Sin.	SIGU	4-Feb-12	Clay	0.75	6–8	6	540.0
9.5.1.2	Rio Bravo, Tam.	TAMPS_21	8-Aug-12	Sandy clay loam	0.81	6–8	8	2592.0
9.5.1.2	Matamoros, Tam.	TAMPS_28	3-Aug-12	Sandy loam	0.76	6–8	8	790.4
10.2.4.1	Buenaventura, Chih.	CHIH_03	7-Aug-12	Clay loam	0.90	8–9	8	1152.0
10.2.4.1	Julimes, Chih.	CHIH_18	2-Aug-12	Sandy clay loam	0.90	8–9	8	1500.0
10.2.4.1	Matamoros, Coah.	LAG_12	10-Aug-12	Sandy clay loam	0.75	8–9	8	398.7
10.2.4.1	Fco. I Madero, Coah.	LAG_09	11-Aug-12	Silty clay	0.65	8–9	9	1657.5
10.2.2.8	Cd. Obregon, Son.	SON_12	12-Oct-12	Clay loam	0.66	3–5	7	2772.0
10.2.2.8	Huatabampo, Son.	SON_02	23-Oct-12	Clay	0.75	3–5	7	2160.0
14.3.1.2	Guasave, Sin.	SIN_72	12-Dec-12	Clay	0.76	6–8	8	820.8
14.3.1.2	Guasave, Sin.	SIN_77	14-Jan-13	Clay	0.76	6–8	8	1860.4
14.3.1.2	Navolato, Sin.	SIN_81	4-Jan-13	Clay	0.80	6–8	8	1052.0
14.3.1.2	Navolato, Sin.	SIN_82	12-Jan-13	Clay	0.80	6–8	8	1071.0
9.5.1.2	Reynosa, Tam.	TAMPS_15	5-Feb-13	Sandy clay loam	0.81	6–8	8	4128.0
9.5.1.2	Rio Bravo, Tam.	TAMPS_21	4-Feb-13	Sandy loam	0.81	6–8	8	3888.0

[a] Ecoregion as described by INEGI-CONABIO-INE (2008): (i) 9.5.1.2 = Tamaulipas coastal plain with xerophile vegetation or without apparent vegetation; (ii) 10.2.2.8 = Floodplains of the Yaqui, Mayo, and Fuerte Rivers with xerophile shrubs and mesquite; (iii) 10.2.3.3 = Floodplains and rolling hills of the Vizcaíno and Magdalena Deserts with xerophile sarco-sarcocrassicaule and halophytic vegetation; (iv) 10.2.4.1 = Central plains of the Chihuahuan Desert with xerophile-halophytic microphyllus vegetation; and (v) 14.3.1.2 = Sinaloa coastal plain with low thorny forest

[b] Site ID was designated by combining the first letters of the state where the trials were conducted or the first two letters of the name of the landowner and the number of the trial at each particular site
[c] Studies were grouped as Experimental or Pilot Phase

Supplementary Table 3 Phenotypic, agronomic, and insect damage characteristics evaluated for MON-89Ø34-3 × MON-88Ø17-3, MON-89Ø34-3 × MON-ØØ6Ø3-6, and MON-ØØ6Ø3-6 GM maize hybrids and their conventional control in Experimental and Pilot Phase studies in Mexico during 2009–2013

Characteristic type	Characteristic	Evaluation timing[a]	Evaluation description
Phenotypic	Seedling vigor	V3	Rating scale 1 to 9, where 1=poor and 9=best
	Days-to-anthesis	VT	Days from planting to 50% pollen shed
	Days-to-silking	R1	Days from planting to 50% silking
	Ear height	R1	Distance from soil surface to ear attachment node (cm)
	Plant height	R2	Distance from soil surface to the flag leaf collar (cm)
Agronomic	Early stand count	V3	Seedling count per unit area[b]
	Final stand count	R6	Plant count per unit area[b]
	Root lodging	R6	% plants leaning >45° at the root crown per unit area[b]
	Stalk lodging	R6	% plants broken below the ear per unit area[b]
	Dropped ears	R6	Number of ears dropped per unit area[b]
	Grain moisture	R6	% grain moisture at harvest
	Grain yield	R6	Grain yield expressed in tons per hectare
Insect damage	Stalk borer (*Diatraea* spp.) —tunnel length	R5–6	Total stalk tunnel length per plant (cm) from 10 plants for Experimental Phase studies and 50 plants for Pilot Phase studies
	Stalk borer (*Diatraea* spp.) —tunnel number	R5–6	Count of total stalk tunnels per plant from 10 plants for Experimental Phase studies and 50 plants for Pilot Phase studies
	Diabrotica root damage	VT–R1	Modified root-node injury scale 0 to 3 (Oleson et al. 2005) from 10 plants for Experimental Phase studies and 50 plants for Pilot Phase studies
	Corn earworm (*Helicoverpa zea* or *Spodoptera* spp.) damage	R2–3	Ear surface damage (cm^2) per plant from 10 plants for Experimental Phase studies and 50 plants for Pilot Phase studies
	Spodoptera leaf damage	V2–12	Leaf damage scale 0 to 9 (Davis et al. 1992) from 10 plants for Experimental Phase studies and 50 plants for Pilot Phase studies
	Cutworm (*Agrotis* or *Spodoptera* spp.) damage[c]	V1–2	Number of seedlings damaged per unit area[b]

[a] Ritchie et al. (2005)
[b] Unit area = 2-row plot for Experimental Phase studies and 50-m-long subsample for Pilot Phase studies
[c] Cutworm (Lepidopteran) damage was evaluated when present

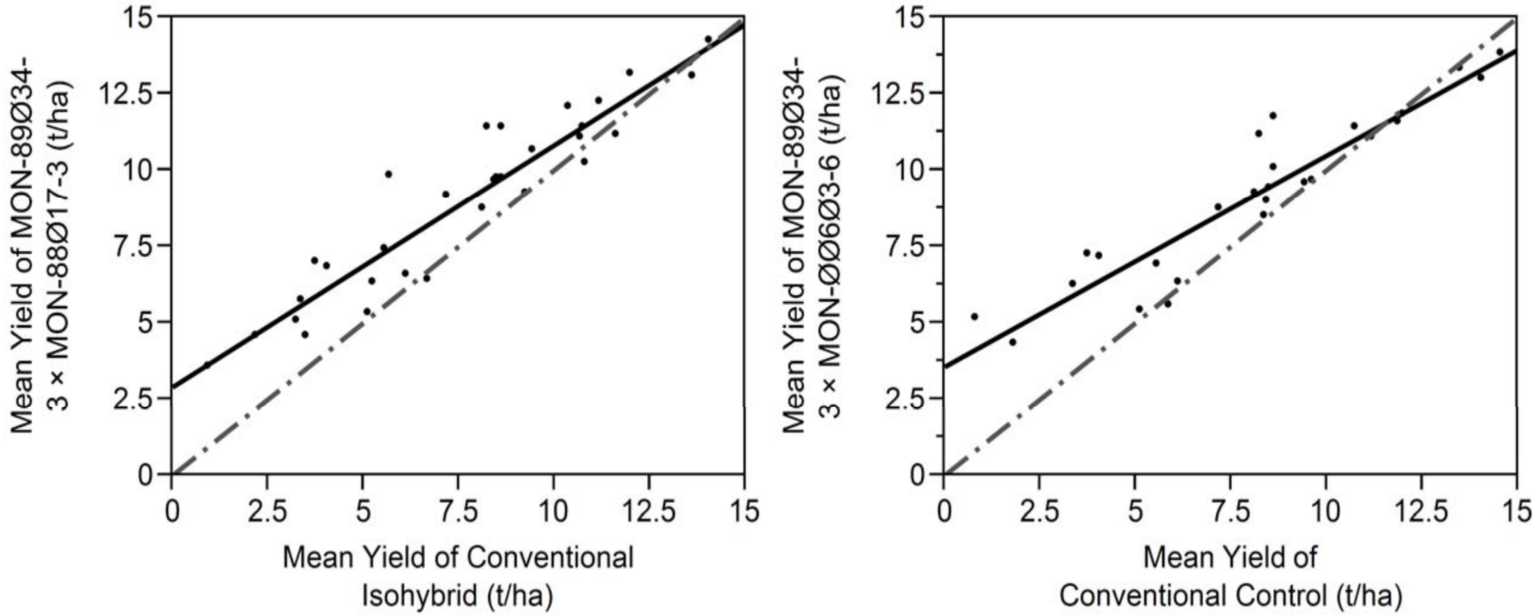

Supplementary Fig. 1 Overall mean yield advantage of MON-89Ø34-3 × MON-88Ø17-3 (left) and MON-89Ø34-3 × MON-ØØ6Ø3-6 (right) GM maize hybrids (*solid lines*) relative to the conventional control (*dashed lines*). Data in each graph are combined across 32 Experimental Phase and 26 Pilot Phase studies

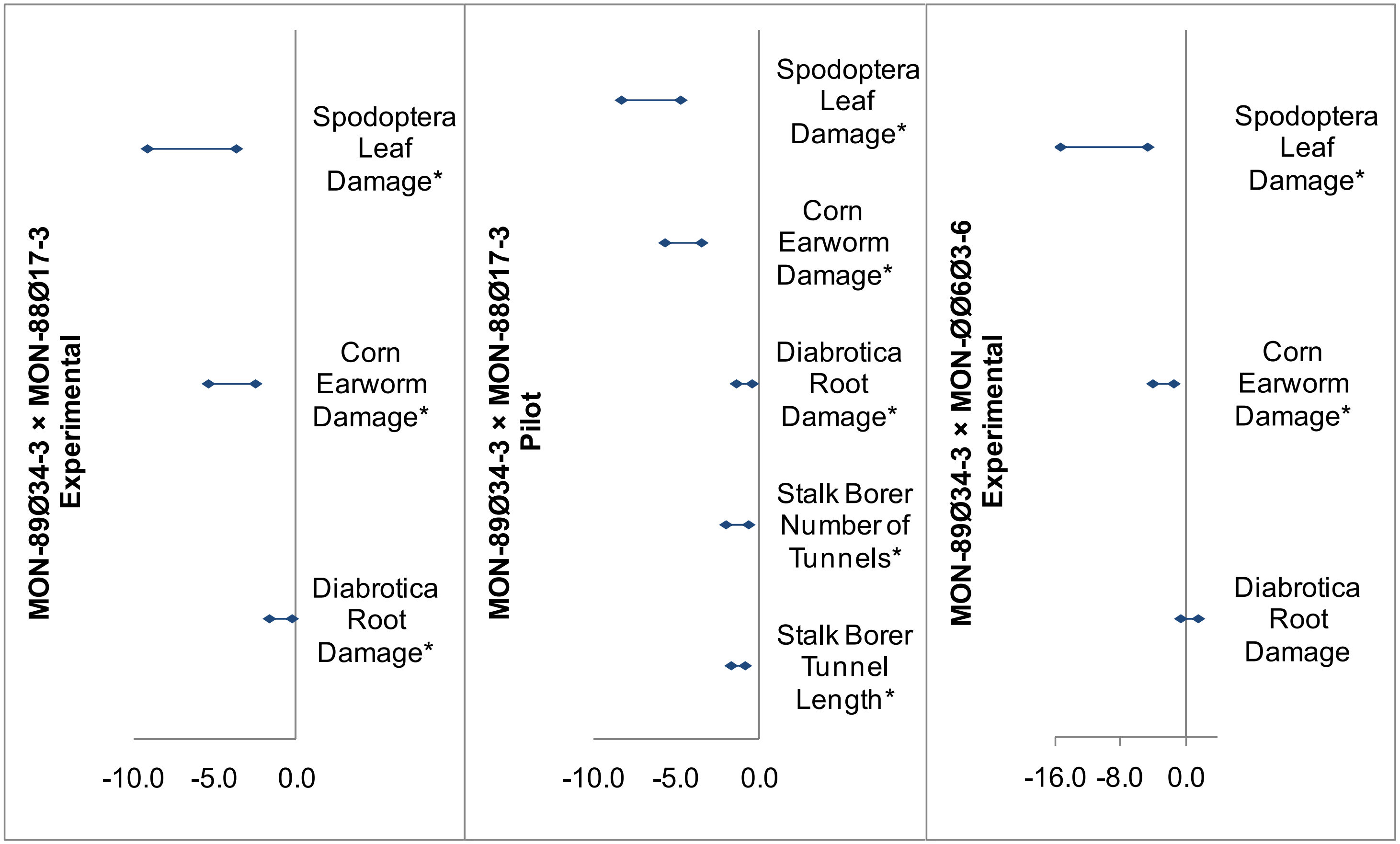

Supplementary Fig. 2 Insect damage combined study effects and confidence intervals for MON-89Ø34-3 × MON-88Ø17-3 and MON-89Ø34-3 × MON-ØØ6Ø3-6 GM maize hybrids compared to the conventional control from Experimental and Pilot Phase studies. Confidence intervals are shown as standardized differences (differences indicated in standard deviation units) derived from the meta-analysis. *Asterisks* indicate statistically significant differences between test and control at the 5% level of significance

References

Davis FM, Ng SS, Williams WP (1992) Visual rating scales for screening whorl stage corn for resistance to fall armyworm. Miss Agric For Exp Stn Tech Bull 186

INEGI-CONABIO-INE (Instituto Nacional de Estadística, Geografía e Informática– Comisión Nacional para el Conocimiento y Uso de la Biodiversidad–Instituto Nacional de Ecología) (2008) 'Ecorregiones Terrestres de México'. Escala 1:1000000. México. Available at: http://www.conabio.gob.mx/informacion/metadata/gis/ecort08gw.xml?_xsl=/db/metadata/xsl/fgdc_html.xsl&_indent=no. Accessed 11 May 2016

Oleson JD, Park Y, Nowatzki TM, Tollefson JJ (2005) Node-injury scale to evaluate root injury by corn rootworms (Coleoptera: Chrysomelidae). J Econ Entomol 98:1–8

Ritchie SW, Hanway JJ, Benson GO (2005) How a corn plant develops. Special Report #48. Iowa State University, Ames, IA
